# A survey study of the association between mobile phone use and daytime sleepiness in California high school students

**DOI:** 10.1186/1471-2458-13-840

**Published:** 2013-09-12

**Authors:** Nila Nathan, Jamie Zeitzer

**Affiliations:** 1 Mountain View High School, Mountain View, 3535 Truman Avenue, Mountain View, CA 94040, USA; 2 Department of Psychiatry and Behavioral Sciences, Stanford University, 3801 Miranda Avenue (151Y), Stanford CA 94305, Palo Alto, CA 94304, USA; 3 Mental Illness Research, Education, and Clinical Center, VA Palo Alto Health Care System, 3801 Miranda Avenue (151Y), Palo Alto, CA 94304, USA

**Keywords:** Adolescent, Sleep deprivation, Mobile phone, Survey

## Abstract

**Background:**

Mobile phone use is near ubiquitous in teenagers. Paralleling the rise in mobile phone use is an equally rapid decline in the amount of time teenagers are spending asleep at night. Prior research indicates that there might be a relationship between daytime sleepiness and nocturnal mobile phone use in teenagers in a variety of countries. As such, the aim of this study was to see if there was an association between mobile phone use, especially at night, and sleepiness in a group of U.S. teenagers.

**Methods:**

A questionnaire containing an Epworth Sleepiness Scale (ESS) modified for use in teens and questions about qualitative and quantitative use of the mobile phone was completed by students attending Mountain View High School in Mountain View, California (n = 211).

**Results:**

Multivariate regression analysis indicated that ESS score was significantly associated with being female, feeling a need to be accessible by mobile phone all of the time, and a past attempt to reduce mobile phone use. The number of daily texts or phone calls was not directly associated with ESS. Those individuals who felt they needed to be accessible and those who had attempted to reduce mobile phone use were also ones who stayed up later to use the mobile phone and were awakened more often at night by the mobile phone.

**Conclusions:**

The relationship between daytime sleepiness and mobile phone use was not directly related to the volume of texting but may be related to the temporal pattern of mobile phone use.

## Background

Mobile phone use has drastically increased in recent years, fueled by new technology such as ‘smart phones’. In 2012, it was estimated that 78% of all Americans aged 12–17 years had a mobile phone and 37% had a smart phone [1]. Despite the growing number of adolescent mobile phone users, there has been limited examination of the behavioral effects of mobile phone usage on adolescents and their sleep and subsequent daytime sleepiness.

Mobile phone use in teens likely compounds the biological causes of sleep loss. With the onset of puberty, there are changes in innate circadian rhythms that lead to a delay in the habitual timing of sleep onset [2]. As school start times are not correspondingly later, this leads to a reduction in the time available for sleep and is consequently thought to contribute to the endemic sleepiness of teenagers. The use of mobile phones may compound this sleepiness by extending the waking hours further into the night. Munezawa and colleagues [3] analyzed 94,777 responses to questionnaires sent out to junior and senior high school students in Japan and found that the use of mobile phones for calling or sending text messages after they went to bed was associated with sleep disturbances such as short sleep duration, subjective poor sleep quality, excessive daytime sleepiness and insomnia symptoms. Soderqvist et al. in their study of Swedish adolescents aged 15–19 years, found that regular users of mobile phones reported health symptoms such as tiredness, stress, headache, anxiety, concentration difficulties and sleep disturbances more often than less frequent users [4]. Van der Bulck studied 1,656 school children in Belgium and found that prevalent mobile phone use in adolescents was related to increased levels of daytime tiredness [5]. Punamaki et al. studied Finnish teens and found that intensive mobile phone use lead to more health complaints and musculoskeletal symptoms in girls both directly and through deteriorated sleep, as well as increased daytime tiredness [6]. In one prospective study of young Swedish adults, aged 20–24, those who were high volume mobile phone users and male, but not female, were at greater risk for developing sleep disturbances a year later [7]. The association of mobile phone utilization and either sleep or sleepiness in teens in the United States has only been described by a telephone poll. In the 2011 National Sleep Foundation poll, 20% of those under the age of 30 reported that they were awakened by a phone call, text or e-mail message at least a few nights a week [8]. This type of nocturnal awakening was self-reported more frequently by those who also reported that they drove while drowsy.

As there has been limited examination of how mobile phone usage affects the behavior of young children and adolescents, none of which have addressed the effects of such usage on daytime sleepiness in U.S. teens, it seemed worthwhile to attempt a cross-sectional study of sleep and mobile phone utilization in a U.S. high school. As such, it was the purpose of this study to examine the association of mobile phone utilization and sleepiness patterns in a sample of U.S. teens. We hypothesized that an increased number of calls would be associated with increased sleepiness.

## Methods

We designed a survey that contained questions concerning sleepiness and mobile phone use (see Additional file 1). Sleepiness was assessed using a version of the Epworth Sleepiness Scale (ESS) [9] modified for use in adolescents [10]. The modified ESS consists of eight questions that assessed the likelihood of dozing in the following circumstances: sitting and reading, watching TV, sitting inactive in a public place, as a passenger in a car for an hour without a break, lying down to rest in the afternoon when circumstances permit, sitting and talking to someone, sitting quietly after a lunch, in a car while stopped for a few minutes in traffic. Responses were limited to a Likert-like scale using the following: no chance of dozing (0), slight chance of dozing (1), moderate chance of dozing (2), or high chance of dozing (3). This yielded total ESS scores ranging from 0 to 24, with scores over 10 being associated with clinically-significant sleepiness [9]. We also included a set of modified questions, originally designed by Thomée et al., that assess the subjective impact of mobile phone use [7]. These included the number of mobile calls made or received each day, the number of texts made or received each day, being awakened by the mobile phone at night (never/occasionally/monthly/weekly/daily), staying up late to use the mobile phone (never/occasionally/monthly/weekly/daily), expectations of accessibility by mobile phone (never/occasionally/daily/all day/around-the-clock), stressfulness of accessibility (not at all/a little bit/rather/very), use mobile phone too much (yes/no), and tried and failed to reduce mobile phone use (yes/no).

An email invitation to complete an electronic form of the survey (http://www.surveymonkey.com) was sent to the entire student body of the Mountain View High School, located in Mountain View, California, USA, on April 5, 2012. Out of the approximately 2,000 students attending the school, a total of 211 responded by the collection date of April 23, 2012. Data analyses are described below (OrginPro8, OriginLab, Northampton MA). Summary data are provided as mean ± SD for age and ESS and as median (range) for the number of texts and/or phone calls made or received per day as these were non-normally distributed (p’s <; 0.001, Kolmogorov Smirnov test). To examine the relationship between sleepiness and predictor variables, stepwise multivariate regression analyses were performed. Collinearity in the data was examined by calculating the Variance Inflation Factor (VIF). *Post hoc* t-tests, ANOVA, Mann–Whitney U tests, and Spearman correlations were used, as appropriate, to examine specific components of the model and their relationship to sleepiness. χ^2^ tests were used to examine categorical variables. The study was done within the regulations codified by the Declaration of Helsinki and approved by the administration of Mountain View High School.

## Results

Sixty-eight males and 143 females responded to the survey. Most (96.7%) respondents owned a mobile phone. The remainder of the analyses presented herein is on the 202 respondents (64 male, 138 female) who indicated that they owned a mobile phone (Tables 1 and 2). The youngest participant in the survey was 14 years old and the oldest was 19 years old (16 ± 1.2 years), representative of the age range of this school. The median number of mobile phone calls made or received per day was 2 and ranged from 0 to 60. The median number of text messages sent or received per day was 22.5 and ranged from 0 to 700. While about half of the respondents (53%) had never been awakened by the mobile phone at night, 35% were occasionally awakened, 5.9% were awakened a few times a month, 5.0% were awakened a few times a week, and 1.0% were awakened almost every night. About one-quarter (27%) of respondents had never stayed awake later than a target bedtime in order to use the mobile phone, however 36% occasionally stayed awake, 19% stayed awake a few times a month, 8.5% stayed awake a few times a week, and 10% stayed awake almost every night in order to use the mobile phone. In regards to feeling an expectation of accessibility, 7.5% reported that they needed to be accessible around the clock, 26% reported that they needed to be accessible all day, 52% reported they needed to be accessible daily, 13% reported that they only needed to be accessible now and then, and 1.0% reported they never needed to be accessible. Nearly half (49%) of the survey participants viewed accessibility via mobile phones to be not at all stressful, 45% found it to be a little bit stressful, 4.5% found it rather stressful, and 1.0% found it very stressful. More than one-third (36%) reported that they or someone close to them thought that they used the mobile phone too much. Few (17%) had tried but were unable to reduce their mobile phone use.

**Table 1:** Descriptive statistics - continuous variables

	**n**	**Age (years)**	**# calls/d**	**# texts/d**	**# calls or texts/d**	**ESS**
All	202	16 ± 1.2	2 (0–60)	22.5 (0–700)	26 (0–702)	6.8 ± 3.5
Male	64	16 ± 1.2	2 (0–40)	10 (0–300)	12 (0–305)	5.6 ± 3.4
Female	138	16 ± 1.2	3 (0–60)	30 (0–700)**	33 (0–702)**	7.4 ± 3.3^††^

Data are shown as mean ± SD (age, ESS), or median (range) (texting/calling). **: p <; 0.01, Mann–Whitney U-test, ^††^: p <; 0.01, t-test.

**Table 2:** Descriptive statistics - categorical variables

**Use phone too much?**	***Yes***	***No***			
All	71	129			
Male	14	48			
Female**	57	81			
**Tried to reduce using phone?**	*Yes*	*No*			
All	34	168			
Male	5	59			
Female*	29	109			
**Awakened by your mobile phone at night?**	*Never*	*Occasionally*	*A few times a month*	*A few times a week*	*Almost every day*
All	108	70	12	10	2
Male	36	24	2	1	1
Female	72	46	10	9	1
**Stayed up late to use cell phone?**	*Never*	*Occasionally*	*A few times a month*	*A few times a week*	*Almost every day*
All	54	72	38	17	20
Male	27	24	8	2	2
Female**	27	48	30	15	18
**Expected accessibility?**	*Never*	*Now and then*, *but not daily*	*Daily*, *but not all day*	*All day*	*Around the clock*
All	2	27	104	53	15
Male	2	10	38	9	4
Female*	0	17	66	44	11
**Perceive accessibility as stressful?**	*Not at all stressful*	*A little bit stressful*	*Rather stressful*	*Very stressful*	
All	99	91	9	2	
Male	38	23	2	1	
Female	61	68	7	1	

*: p <; 0.05, **: p <; 0.01, *χ*^2^ test between males and females.

Subjective sleepiness on the ESS ranged from 0 to 18 (6.8 ± 3.5, with higher numbers indicating greater sleepiness), with 25% of participants having ESS scores in the excessively sleepy range (ESS ≥ 10). We examined predictors of subjective sleepiness (ESS score) using stepwise multivariate regression analysis with the following independent variables: age, sex, frequency of nocturnal awakening by the phone, frequency of staying up too late to use the phone, self-perceived accessibility by phone, stressfulness of this accessibility, attempted and failed to reduce phone use, excessive phone use determined by others, number of texts per day, and number of phone calls per day. Only subjects with complete data sets were used in our modeling (n = 191 of 202). Our final model (Table 3) indicated that sex, frequency of accessibility, and a failed attempt to reduce mobile phone use were all predictive of daytime sleepiness (F_6,194_ = 4.35, p <; 0.001, r^2^ = 0.12). These model variables lacked collinearity (VIF’s <; 3.9), indicating that they were not likely to represent the same source of variance. Despite the lack of significance in the multivariate model, given previously published data [4-6], we independently tested if there was a relationship between the number of estimated texts and sleepiness, but found no such correlation (r = 0.13, p = 0.07; Spearman correlation). In examining the final model, it appears that those who felt that they needed to be accessible “around the clock” (ESS = 9.2 ± 2.9) were sleepier than all others (ESS = 6.7 ± 3.4) (p <; 0.01, *post hoc t*-test). The relationship between sleepiness and reporting having tried, but failed, to reduce mobile phone use was such that those who had tried to reduce phone use were more sleepy (ESS = 8.3 ± 3.6) than those who had not (ESS = 6.5 ± 3.4) (p <; 0.01, *post hoc t*-test). While more females had tried to reduce their mobile phone use, sex did not modify the relationship between the attempt to reduce mobile phone use and sleepiness (p = 0.32, two-way ANOVA), thus retaining attempt and failure to reduce mobile phone use as an independent modifier of ESS scores.

**Table 3:** Multivariate model

	**Value**	**SE**	**t-value**	**p-value**
*Intercept*	9.05	0.860	10.5	<;0.0001
*Sex*	0.798	0.258	3.09	<;0.01
*Accessible* - *never*	−4.09	2.49	−1.64	0.10
*Accessible* - *now and then*	−1.98	1.07	−1.85	0.066
*Accessible* - *daily*	−2.04	0.910	−2.24	<;0.05
*Accessible* - *all day*	−2.58	0.958	−2.69	<;0.01
*Reduce use*?	0.659	0.317	2.08	<;0.05

Final model predicting ESS. Sex was coded as male = −1, female = 1; For accessibility, “Accessible – around the clock” was used as a reference category and each category was scored as yes = 1, blank = 0; Reduce use? was coded as yes = 1, no = −1.

In an attempt to better understand the relationship between ESS and accessibility, we parsed the population into those who felt that they needed to be accessible around the clock (7.4%) and those who did not (92.6%). The most accessible group, as compared to the less accessible group, had a numerically though not statistically significantly higher texting rate (50 vs. 20 per day; p = 0.07, Mann–Whitney *U* test), but were awakened more at night by the phone (27% vs. 4%, weekly or daily; p <; 0.05, χ^2^ test), and stayed awake later than desired more often (40% vs. 17%, weekly or daily; p <; 0.05, χ^2^ test). We did a similar analysis, parsing the population into those who had attempted but failed to reduce their use of their mobile phone (17%) with those who had not (83%). Those who had attempted to reduce their mobile phone use had a higher texting rate (60 vs. 20 per day; p <; 0.01, Mann–Whitney U test) and stayed awake later than desired more often (53% vs. 11%, weekly or daily; p <; 0.01, χ^2^ test), but were not awakened more at night by the phone (12% vs. 5%, weekly or daily; p = 0.26, χ^2^ test).

## Discussion

Given previous research on the topic, our *a priori* hypothesis was that teenagers who use their phone more often at night are likely to be more prone to daytime sleepiness. We did not, however, observe this simple relationship in this sample of U.S. teens. We did find that being female, perceived need to be accessible by mobile phone, and having tried but failed to reduce mobile phone usage were all predictive of daytime sleepiness, with the latter two likely being moderated by increased use of the phone at night. Previous work has shown that being female was associated with higher ESS scores [11]. It may be that adolescent females score higher on the ESS without being objectively sleepier, though this remains to be tested. Our analyses revealed that staying up late to use the mobile phone and being awakened by the mobile phone may be involved in the relationship between increased ESS scores and perceived need to be accessible by mobile phone and a past attempt to decrease mobile phone use. These analyses reveal some of the complexity of assessing daytime sleepiness, which is undoubtedly multifactorial. If the sheer number of text messages being sent per day is directly associated daytime sleepiness, it is likely with a small effect size. Our work, of course, is not without its limitations. Data were collected from a sample of convenience at a single, public high school in California. Only 10% of students responded to the survey and this may have introduced some response bias to the data. The data collected were cross-sectional; a longitudinal collection would have enabled a more precise analysis of moderators and mediators as well as a more accurate interpretation of causal relationships. Also, we did not objectively record the number of texts, so there may be a certain degree of bias or uncertainty associated with self-report of number of texts and calls. Several variables that might influence sleepiness both directly and indirectly through mobile phone use (e.g., socioeconomic status, comorbid sleep disorders, medication use) were not assessed. Future studies on the impact of mobile phone use on sleep and sleepiness should take into account the multifactorial and temporal nature of these behaviors.

## Conclusions

The endemic sleepiness found in adolescents is multifactorial with both intrinsic and extrinsic factors. Mobile phone use has been assumed to be one source of increased daytime sleepiness in adolescents. Our analyses revealed that use or perceived need of use of the mobile phone during normal sleeping hours may contribute to daytime sleepiness. As overall number of text messages did not significantly contribute to daytime sleepiness, it is possible that a temporal rearrangement of phone use (e.g., limiting phone use during prescribed sleeping hours) might help in alleviating some degree of daytime sleepiness.

## Abbreviations

ESS: Epworth sleepiness scale; SD: Standard deviation; ANOVA: Analysis of variance.

## Competing interests

The authors declare that they have no competing interests.

## Authors’ contributions

JMZ and NN designed the study, analyzed the data, and drafted the manuscript. Both authors have read and approved the final manuscript.

## Pre-publication history

The pre-publication history for this paper can be accessed here:

http://www.biomedcentral.com/1471-2458/13/840/prepub

## Supplementary Material

Additional file 1 Questionnaire. Click here for file
