# Supplementary material for: A survey study of the association between mobile phone use and daytime sleepiness in California high school students
Source: BMC Public Health. 2013 Sep 12;13:840. doi: 10.1186/1471-2458-13-840 (PMC3847218; doi:10.1186/1471-2458-13-840)
Supplement: Additional file 1 — Questionnaire. [file 1471-2458-13-840-S1.doc]

1. Age

*OPEN ENDED*

1. Sex

*MALE/FEMALE*

1. Do you own a cell phone?

*YES/NO*

**FOR ALL QUESTIONS, PLEASE REFER TO YOUR IMPRESSIONS ABOUT THE LAST MONTH**

1. On average, how many mobile phone calls have you made or received per day?

*OPEN ENDED*

1. On average, how many text messages have you made or received per day?

*OPEN ENDED*

1. How often have you been awakened by your mobile phone at night?

*Never*

*Occasionally*

*A few times a month*

*A few times a week*

*Almost every day*

1. How often have you stayed up later than you wanted in order to talk or text on your cell phone?

*Never*

*Occasionally*

*A few times a month*

*A few times a week*

*Almost every day*

1. To what extent are you expected by those around you to be accessible via the mobile phone?

*Never*

*Now and then, but not daily*

*Daily, but not all day*

*All day*

*Around the clock*

1. To what extent do you perceive accessibility via mobile phones as stressful?

*Not at all stressful*

*A little bit stressful*

*Rather stressful*

*Very stressful*

1. Do you or someone close to you think that you use the mobile phone too much?

*YES/NO*

1. Have you tried, but failed, to cut down on your use of the mobile phone?

*YES/NO*

1. What is you chance of dozing in the following circumstances:

Sitting and reading

*No chance of dozing*

*Slight chance of dozing*

*Moderate chance of dozing*

*High chance of dozing*

1. Sitting inactive in a public place (e.g a theater or a meeting)

*No chance of dozing*

*Slight chance of dozing*

*Moderate chance of dozing*

*High chance of dozing*

1. In a car for an hour without a break

*No chance of dozing*

*Slight chance of dozing*

*Moderate chance of dozing*

*High chance of dozing*

1. Lying down to rest in the afternoon when circumstances permit

*No chance of dozing*

*Slight chance of dozing*

*Moderate chance of dozing*

*High chance of dozing*

1. Sitting and talking to someone

*No chance of dozing*

*Slight chance of dozing*

*Moderate chance of dozing*

*High chance of dozing*

1. Sitting quietly after a lunch

*No chance of dozing*

*Slight chance of dozing*

*Moderate chance of dozing*

*High chance of dozing*

1. In a car, while stopped for a few minutes in traffic

*No chance of dozing*

*Slight chance of dozing*

*Moderate chance of dozing*

*High chance of dozing*

Cell phone questions adapted from: Thomée et al. (2011) Mobile phone use and stress, sleep disturbances, and symptoms of depression among young adults - a prospective cohort study. BMC Public Health 11:66

Sleep questions from a modified version of the Epworth Sleepiness Scale: Melendres et al. (2004) Daytime sleepiness and hyperactivity in children with suspected sleep-disordered breathing. Pediatrics 114:768-775.
